# Biportal endoscopic spine surgery for cervical disk herniation: A technical notes and preliminary report

**DOI:** 10.1097/MD.0000000000029751

**Published:** 2022-07-08

**Authors:** Seok Bong Jung, Nackhwan Kim

**Affiliations:** a Spine Center, Jinju Bon Hospital, Jinju-si, Gyeongsangnam-do, Republic of Korea; b Department of Physical Medicine and Rehabilitation, Korea University Ansan Hospital, Ansan-si, Gyeonggi-do, Republic of Korea.

**Keywords:** biportal endoscopy, cervical disk herniation, cervical discectomy, decompression

## Abstract

Biportal endoscopic spine surgery (BESS) for cervical disk herniation (CDH) has been rarely reported. The aim of the article is to describe a novel BESS as a posterior approach for CDH and report the preliminary outcomes and complications. This single-centered retrospective chart review included 109 consecutive patients who underwent BESS for symptomatic single-level CDH. Working and viewing portals were created in each unilateral paravertebral area at the target disk level. Endoscopic exploration allowed for effective and minimally invasive decompression via safe access to the medial foramen with minimal laminectomy and facetectomy. Clinical outcomes, including the visual analog scale, neck disability index, Macnab criteria, and the motor function of the involved arm, were evaluated at 4, 8, 12, and 24 postoperative weeks. Visual analog scale and neck disability index improved significantly at 24 weeks postoperatively (*P* < .01). According to the Macnab criteria, “excellent,” “good,” and “fair” results were obtained for 55.9%, 30.3%, and 13.8% of patients, respectively. The post 24-week distribution of the involved upper extremity strength grade was significantly improved compared to the initial value (*P* = .02). One patient had a motor weakness with a decreased grade over 4 weeks from excessive irrigation. The posterior approach of BESS was efficient and feasible for the treatment of CDH.

## 1. Introduction

Anterior cervical discectomy and fusion (ACDF) has long been the standard surgical strategy for cervical disk herniation (CDH) treatment.^[1–5]^ However, significant complications associated with this surgical approach have led to a demand for safer and less invasive alternatives. Endoscopic discectomy through a single portal aims to preserve disk function by resecting only a small amount of the tissue irritating the neural elements, with reportedly good to excellent outcomes and low complication rates in patients with CDH.^[6–9]^

Recently, attempts have been made to increase the efficiency of endoscopic decompression. In particular, biportal endoscopic spine surgery (BESS) has been reported as a treatment strategy for lumbosacral disk herniation and stenosis that provides a more flexible field of view and greater degrees of freedom than the uniportal endoscopic approach.^[10]^ The posterior approach of BESS has been the focus of several articles, but most publications have reported on the approach as a strategy for treating lumbosacral lesions. No attempt has been made to report its usefulness in treating cervical lesions.

This study aimed to describe the surgical technique and investigate the clinical outcomes associated with BESS as an intervention for CDH.

## 2. Materials and Methods

This retrospective study was conducted in compliance with the Declaration of Helsinki and was approved by the institutional review board. Informed consent was provided by each patient.

### 2.1. Patient population

We performed a retrospective review of 109 patients who underwent BESS for symptomatic CDH at a single center between February 2018 and July 2020. Only patients who had radicular symptoms for at least 3 months and had undergone conservative management for at least 1 month were considered for cervical BESS. Symptomatic single-level disks were identified using magnetic resonance imaging (MRI) and clinically concordant signs. We excluded patients who had disk protrusion without radicular pain, herniation of ≥2 levels for which diagnostic significance could not be distinguished, accompanying chronic discogenic pain or segmental instability, a history of significant trauma (eg, traffic accident, severe fall) that could affect recovery from cervical spine disorders, and coexisting pathologic conditions such as infection or malignancy.

### 2.2. Evaluation

Data collection also confirmed that the patients consented to undergo the procedure. The captured data included basic epidemiologic information, x-ray and MRI findings, clinical data, and functional assessment results. Postoperative MRI or computed tomography was performed within 72 hours postoperatively to assess soft tissue complications, such as hematoma formation or edema, and to determine the adequacy of tissue removal. A neuroradiologist independently analyzed and compared the preoperative and postoperative images in terms of the extent of herniation, epidural hematoma formation, spinal nerve edema status and severity, and relevant bony structures. Visual analog scale (VAS) pain scores, neck disability index (NDI) values,^[11]^ Macnab criteria, and manual muscle examination results were assessed preoperatively and at follow-up visits scheduled at 4, 8, 12, and 24 postoperative weeks. Muscle power was assessed using the Medical Research Council (MRC) scale.

### 2.3. Statistical analysis

We conducted a repeated-measures analysis of variance to analyze clinical outcomes, and we compared the periodic outcomes using paired *t tests* at a significance level of .05. Sphericity test of Mauchly and compound symmetry were performed in advance. We further performed post hoc comparisons, and *P* value of <.05 was considered statistically significant. Data analyses were conducted using IBM SPSS Statistics for Windows, version 22.0 (IBM Corp., Armonk, NY).

### 2.4. Surgical Technique

One surgeon (S.B.J) performed all the operations. The conceptual flow of the surgical procedure is as follows: preparation, posterior access, biportal approach, exfoliation and osteotomy, repositioning of neurovascular structures, removal of herniated tissue, exploration and debridement, and closure. The equipment used in the surgery included standard laminectomy instruments (such as hook dissectors, 2-mm-width Kerrison punches, and pituitary forceps), 30-degree 4-mm-diameter arthroscope (Conmed Linvatec^®^), bipolar radiofrequency probe (Trigger-Flex^®^, Ellman International, Inc.), 3.5-mm spherical bur (Conmed Linvatec^®^), and a pressure pump irrigation system (Smith & Nephew, Inc.).

**Figure 1.**
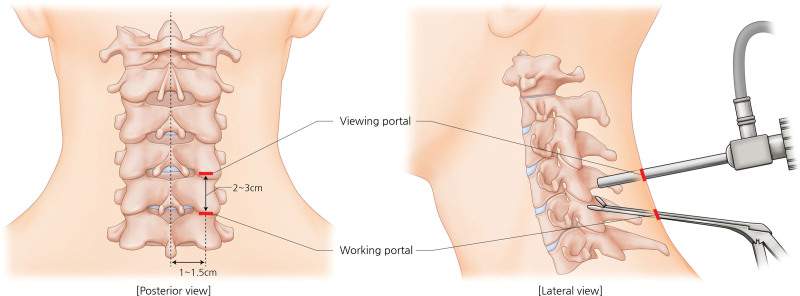
Overview of biportal endoscopic spine surgery for cervical disk herniation (target disk: C4/5). A viewing portal is placed on the cranial side, and a working portal is placed 2 to 3 cm caudally from the viewing portal. The posterior view on left side and lateral view on right side show the relative placement of the 2 portals.

Under general anesthesia with monitoring of vital signs and neurovascular damage, patients were positioned in the standard prone position on a face cradle pad. During preparation, the cervical spine was positioned anatomically and was not flexed or extended. Both the humerus and scapula were gently displaced downward to obtain a lateral fluoroscopic image of the entire cervical spine. The surgical landmarks were identified using metal probes under fluoroscopic guidance. A cautious small skin incision was applied about 1 to 1.5 cm ipsilateral to the spinous process line at the level of the target interlaminar space. Another skin incision was applied 2 to 3 cm below the first incision. The incisions were extended transversally by approximately 1 cm (Fig. 1).

The 2 portals are the viewing and working portals, with the working portal located distally to minimize the extent of laminectomy required. For the same reason, the handle of the working device was positioned in the left hand for right-sided cervical lesions and in the right hand for left-sided lesions. The proximal portal (the viewing portal) was initially extended to the depth of the bony lamina using long Kelly forceps, and a clear endoscopic view was secured through the gentle indwelling of the scope with a continuous irrigation system controlled to a set pressure of <30 mm Hg. The pressure delays the microbleeding time of the surgical space, leading to the hemostasis, and prevents visual disturbance under the scope. The working device (forceps) was inserted and placed within the view.

**Figure 2.**
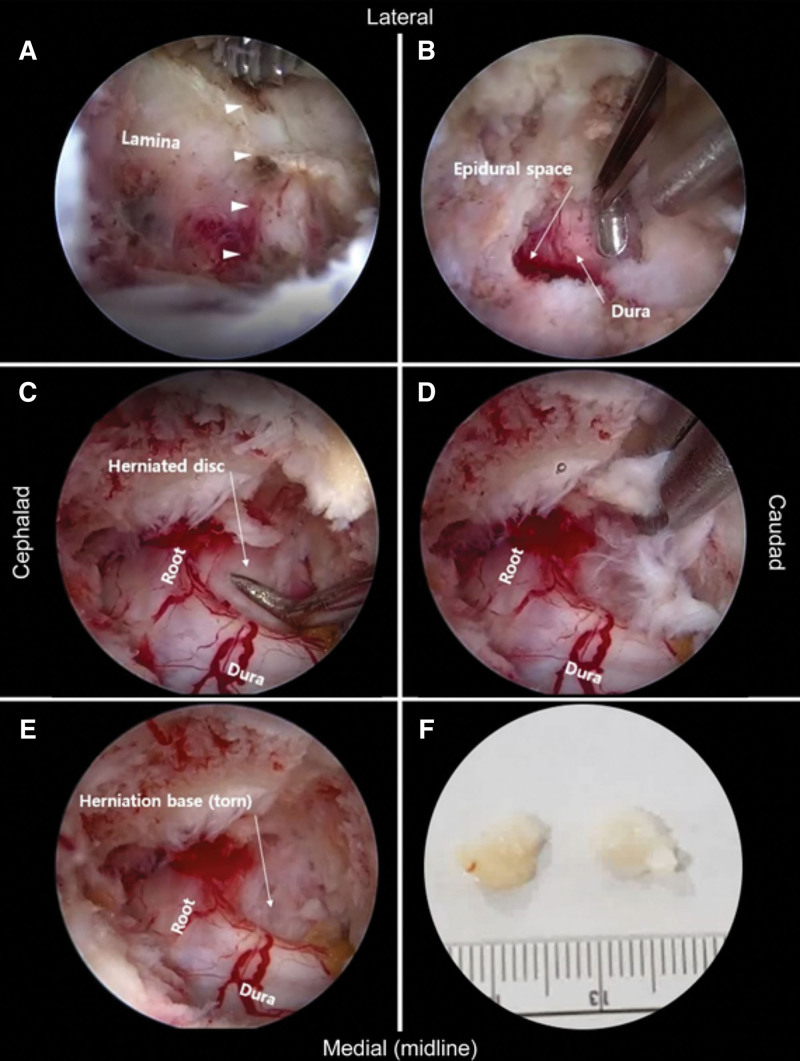
Endoscopic views for approach to the target: osteotomy, repositioning of neurovascular structures, and removal of herniated tissue (a case of right C6/7 subarticular extrusion). After ablating the partial lamina and facet joint by the automated bur and Karrison punch, the dura mater is exposed (A and B). The small arrowheads indicate the V point. A posterolateral portion of C6/7 disk (herniated area) was detected by the indicator tip and removed gently by microforceps (C and D). The base of the removed tissue (E) is observed, and compared to panel C; the dura and sleeve seem to be deviated laterally and caudally. The removed disk materials are measured (F).

After gentle exfoliation of the paraspinal muscles, a 30-degree 4-mm-diameter arthroscope at the proximal portal was positioned at the V point (composed of the superior margin of the caudal lamina, the inferior margin of the cranial lamina, and the starting point of the facet joint). A partial hole was made by manipulating the bur very carefully in the target interlaminar area. The adjacent laminar bone and ligamentum flavum directly underneath were removed bit-by-bit using a 2-mm-width Kerrison punch until the foraminal space and the dural sleeve were identified. A medial facetectomy was then performed using the Kerrison punch (Fig. 2A, B). At this stage, the nerve root, dural sleeve, endplates, and annulus of the target disk were observed (Supplemental Video 1, http://links.lww.com/MD/G797).

The nerve roots are located above the pedicle, and the tilting endoscopic views can be identified from the medial side of the pedicle to the posterolateral aspect of the vertebral body. As the approach progresses, the epidural fat is removed and the dural sleeve and nerve root are revealed. At this time, since the window of exposure is narrow, it may be difficult to distinguish between nerve root and annulus. The 2 tissues were distinguished by checking the subtle mobility or firm end-feeling through an indicator. The tip of the working instrument approached the bottom of the endoscopic view and faced toward the center. The retracted spinal nerve was observed because the herniated tissue was usually located on the ventral side of the root (Fig. 2C). For this reason, the herniated disk tissue was removed using pituitary forceps after gently displacing the nerve using a fine and blunt tool (Fig. 2D). After removing the herniated disk tissue (Fig. 2F), free and flexible movement of the nerve root was confirmed (Fig. 2E). Additional annuloplasty was performed using a radiofrequency probe adjacent to the perforated disk hole. The medial foramen and ventral epidural space were carefully investigated by a blind indicator, and the large wandering tissue or ragged margin of the resected annulus were debrided or irrigated. Usually, venous bleeding from the outer annulus was observed after tissue removal, and bone wax was applied (Supplemental Video 2, http://links.lww.com/MD/G798).

After turning off the perfusion pressure of the irrigation system, the bleeding spot was closely investigated, and the instruments were removed from the 2 portals. After removing the instruments and infused saline, the subcutaneous fascia and skin were sutured.

### 2.5. Postoperative care

The serial neurological evaluation was applied after surgery, and attention was paid to early complications. After 12 hours, sitting was allowed with a rigid collar, and if there was no problem with sitting balance, the patient tried to walk under supervision. The patients were discharged at 2 or 3 days after surgery if their mobility was secured without complications.

## 3. Results

### 3.1. Demographic characteristics

A total of 109 consecutive patients were included in the analysis: 84 men and 25 women with a median age of 52 (range, 34–78; mean: 54.5) years. The median duration of symptoms was 24.0 (range, 6–84) months. The levels of the target disks were C3/4 (n = 4), C4/5 (n = 12), C5/6 (n = 41), C6/7 (n = 45), and C7/T1 (n = 7) (Table 1).

**Table 1.** Patients’ baseline characteristics (n = 109).

**Variables**	**Number**
Sex	
M:F	84:25 (M, 77.1%)
Age (y)	
Mean ± SD	54.5 ± 9.2
Range	34–78
Symptom duration (mo)	
Mean ± SD	22.8 ± 14.2
Range	6–84
Herniation levels, (n (%)	
C3/4	4 (3.7)
C4/5	12 (11.0)
C5/6	41 (37.6)
C6/7	45 (41.3)
C7/T1	7 (6.4)

M = male, F = female, SD = standard deviation.

### 3.2. Perioperative data

The median operative time was 45 (range, 31–75) minutes. The first 15 cases took approximately 75 minutes each, and the most recent 15 cases took approximately 35 minutes each. The surgeons’ skill and proficiency gradually proceeded. The median length of hospitalization was 4 days (range, 2–9). Bleeding was effectively controlled by continuous irrigation and electrocoagulation under endoscopic guidance. On average 3120 mL of saline solution (range, 2500–4500) was used for irrigation, and the estimated blood loss was 230 mL on average (range, 160–340), associated with serum hemoglobin reductions of 1.2 g/dL on average (range 1.0–1.5), immediately after surgery. A natural drainage system was used to drain a mean volume of 42 mL over 3 days postoperatively.

**Figure 3.**
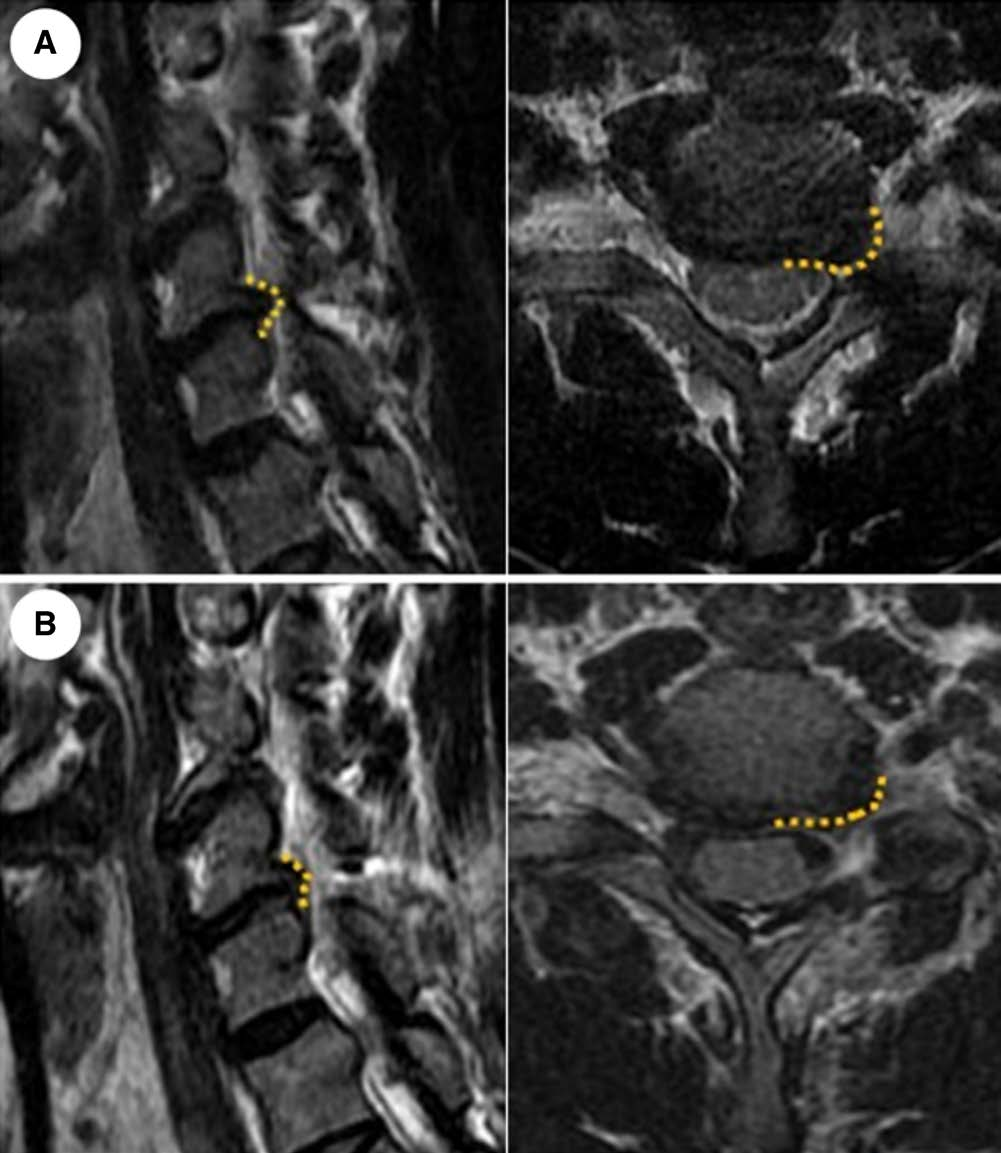
Preoperative and postoperative images. Preoperative MRIs show C6/7 disk protrusion on the left subarticular zone, and the same levels of 1 d postoperative images show a relatively widened space on the zone and removed herniated disk with partial laminectomy and facetectomy. The yellow dashed lines present the annular outline of the target disk. MRI = magnetic resonance imaging.

Preoperative and postoperative images were analyzed, and attenuated disk herniation was reported in all patients (Fig. 3). Imaging analysis yielded no specific evidence of hematoma in the adjacent regions or epidural space around the target disks, spinal nerve or cord edema, or abnormalities of remnant bony structures. Over 6 months, no radiographic progression of adjacent disk degeneration was observed. Cervical flexion and extension limitations and the associated features of clinical instability were not visualized.

### 3.3. Clinical outcomes

Following the procedure, we obtained serial follow-up clinical data for all patients at 4, 8, 12, and 24 weeks. Serial VAS and NDI assessments indicated statistically significant improvements in pain and disability (*P* < .01). The average reported neck pain intensity measured using the VAS was 6.6 ± 2.1 and 1.1 ± 0.8 before and 24 weeks after the procedure, respectively (*F* (1.80, 113.4) = 693.8, *P* < .001). The VAS of arm pain reduced from 7.2 ± 2.4 to 1.0 ± 0.7 (*F* (1.65, 103.7) = 711.0, *P* < .001). The NDI improved from 43.8 ± 15.3 to 6.1 ± 5.5% (*F* (1.20, 75.8) = 243.5, *P* <.001).

**Figure 4.**
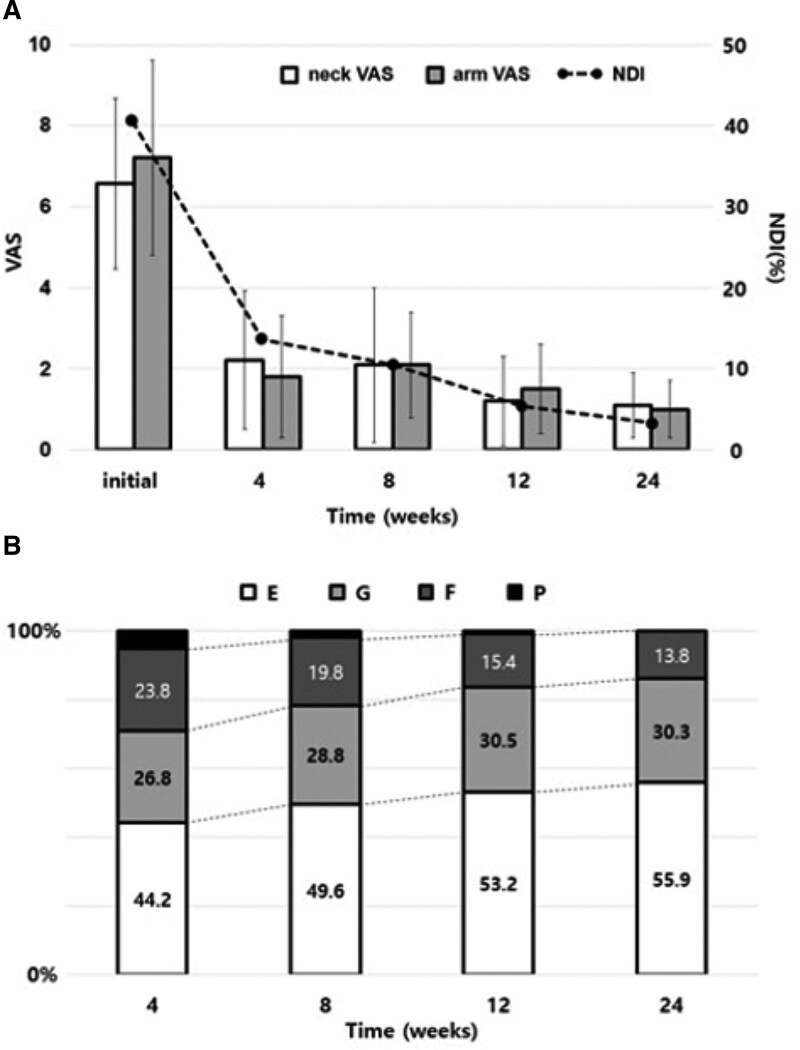
Clinical outcomes. (A) Changes in the VAS pain scores regarding posterior neck and upper extremity pain with NDI improvement. The follow-up VAS and NDI values show a statistically significant improvement (*P* < .01). (B) Postoperatively, patient satisfaction, according to the modified Macnab’s criteria, significantly improved over time (*P* < .01). NDI = neck disability index, VAS = visual analog scale.

Subjective patient satisfaction with the surgery, according to the Macnab criteria, was reported by 55.9% of patients as “excellent,” 30.3% as “good,” and 13.8% as “fair” at 24 postoperative weeks (*P* < .01). The functional loss of muscle strength on the affected side significantly improved in most of the patients (*P <* .02): initially, 64.3% of patients were rated as “normal,” 26.1% as “good,” and 9.6% as “fair;” subsequently, 86.2% were rated as “normal,” 11.9% as “good,” and 1.9% as “fair” (Fig. 4).

There were no serious complications, such as uncontrolled bleeding, vocal change, swallowing difficulty, unpredictive bone injury, or long tract signs. There were no secondary complications, such as infections, spondylitis, vertigo, or thrombosis, during the 24-week follow-up period. In 1 case, postoperative motor weakness of shoulder abduction and elbow flexion was rated as MRC grade 2 from an initial rating of grade 4. Postoperative MRI confirmed longitudinal fluid retention in the dorsal epidural space of the patient’s cervical spine. The patient improved to grade 3 after 3 days and to grade 4 after 4 weeks and showed no abnormalities on follow-up electromyography (4 weeks postoperatively). None of the 109 patients experienced recurrence or subsequent reoperation during the 24-week follow-up period.

## 4. Discussion

Although the gold standard surgical technique for degenerative cervical disease is ACDF, most cervical radicular symptoms caused by CDH can be treated with endoscopic procedures using a single working portal, and the surgical indications are increasing due to the development of new technology and devices. Many reports suggest better outcomes, surgical advantages, and lower complication rates of this approach compared with ACDF.^[12–16]^

Posterior cervical discectomy is developing in the form of minimal invasion. The early proposed “Keyhole” approach method is efficient in posterior approach for preserving bony elements.^[17]^ Furthermore, the disadvantages of the following ACDFs can be effectively avoided: approach-related morbidity, adjacent segment degeneration, implant-related complications, and pseudoarthrosis. Nevertheless, it has been reported that the rate of reoperation increases significantly over time compared to ACDF.^[18]^ Recent studies have reported no statistically significant difference in the incidence of reoperation and complications in long-term follow-up of posterior and anterior approaches, with an average rate of 6% for reoperation, higher than 3.9% for anterior approaches. The technical limitations of the posterior approach are estimated to be narrow vision and the resulting insufficient decompression. This is also related to the fact that in most studies, the cause of reoperation was suggested as the continuation or recurrence of the existing radial pain.^[19]^

Despite minimizing the disadvantages of the conventional procedure, there are several disadvantages to single-portal endoscopic surgery.^[20]^ A recent review summarized the following 4 shortcomings: it is associated with technical challenges and is difficult to learn; there are relatively few practical indications, and the actual indications for endoscopic surgery may be limited to soft CDH or focal stenosis lesions; saline irrigation or hydraulic effects of unrecognized dural damage can occur during surgery; fluoroscopic guidance is required for several processes in the procedure.^[21]^ All 4 disadvantages are consequential in terms of complex manipulation and delayed surgical time in a narrow surgical field. From the surgeon’s point of view, the limited degrees of freedom of the working device may be a practical hurdle in relation to these disadvantages.

The independent approach through 2 portals and the increased degree of freedom of working device have a clear advantage in herniated disk resection. If the accessible space is very narrow, the foraminal ligaments attached to the pedicle must be removed as much as possible. In a single-portal method, the approach direction of chisel and burr is vertical, so anatomical correlation of invisible structures becomes very important.^[22]^ The biportal technique is relatively advantageous for accessing and removing the bony spur because the working device can be accessed from the outside of the scopic view. Therefore, in the case of hard CDH, sufficient decompression by the biportal approach is proposed. The patients in this study had a conservative treatment period of at least 3 months and showed an average symptom duration of 22 months, so it is highly likely that a significant number of patients had hard CDH. Nevertheless, the positive outcome and low recurrence rate may be due to the aforementioned advantages. These results are comparable to those of uniportal approach.^[23]^

Two-portal systems incorporate technical advantages while providing ample space in the hands of the surgeon. There are many advantages gained by separating the field of view and working devices, especially in terms of tissue removal, in that the exploration range is expanded compared with what can be achieved with a single-portal system. This increases the possibility of complete removal of the target tissue, thus broadening the range of indications for the location and type of herniation. Additionally, independent control of the 30-degree endoscope can provide sufficient information to reduce the need for fluoroscopic guidance without unnecessary device location changes.

BESS can be advantageous in terms of minimal bony excision, increased degrees of freedom for working devices, sufficient viewing area, effective decompression, low infection rate, and short morbidity.^[10]^ The principle of symptomatic improvement is decompression of the epidural or foraminal space. This approach attempts efficient decompression by partial laminectomy or facetectomy simultaneously by removing the accessible herniated disk. Therefore, this decompression technique may minimize the amount of bony resection in the approach of ipsilateral subarticular or intraforaminal CDH causing cervical radicular pain and can theoretically be applied to nondiscogenic foraminal stenosis.

According to a retrospective report by the surgeon, the most time-consuming process in the early experiences was to secure the endoscopic view clearly. If the outflow of the irrigation saline through the portals was not appropriate, the quality of the endoscopic view field was poor. The appropriately loose-fitting portals save the surgical time significantly. In addition, it is important to search quickly for the atomic landmark during approach, and the time required to detect V points gradually decreases as the cases increase.^[22]^ Additionally, it is advantageous to shorten the surgical time to set the range of preoperative partial facetectomy.

In 1 case, MRC grade 1 proximal upper extremity weakness persisted for approximately 2 weeks postoperatively. The pattern of weakness did not follow the pattern of myotome, the long tract sign was unclear, and there were no accompanying sensory changes. On MRI at 2 days after surgery, fluid retention in the posterior epidural space was observed from the mid-cervical to the mid-thoracic spine, which was considered to be due to excessive irrigation. As with other endoscopic spine surgery techniques, the perfusion pressure of the irrigation pump can cause nerve compression and epidural hematoma formation; thus, it is vital to check whether the drainage from the working portal is continuous and prevent pressure from increasing in the epidural space. The patient recovered his original muscle strength after 4 weeks, and the pain significantly improved. In a recent study of cervical epidural pressure measured during lumbar BESS, the BESS system was more advantageous for efficient irrigation because of the separated outlet, but maintaining low irrigation pressures of approximately 30 mm Hg, neurological monitoring to avoid epidural fluid retention, and postoperative drainage through a portable wound suction device were recommended.^[24]^

Another disadvantage of this procedure is its relatively high degree of difficulty and the long time required for the operator to gain proficiency. If the surgeon is unfamiliar with the procedure, the incidence of complications, such as dural injury, increases, and anesthesia time is prolonged. However, in the lumbar spine, the overall rate of complications during the initial learning period has been reported to be as high as 10%, with most of them occurring among the first 30 cases.^[25]^

Additional considerations related to the surgical technique are as follows: the posterior neck muscles run parallel to the vertebral body so that the transverse skin incision makes it easier to move; excessive facet joint removal may cause mechanical pain due to fracturing of the remaining joint or cervical instability; and the anatomical structure of the upper cervical spine, including cervical intervertebral disks 4/5, may make it difficult to remove the disk tissue because the nerve roots are just above the pedicle.^[26]^

In conclusion, BESS for the cervical spine is associated with favorable outcomes. However, the study findings should be interpreted with care due to the relatively small sample size and short follow-up period. Symptom improvement was not inferior to that associated with ACDF; there were few complications, and quick recovery was expected. However, this procedure is challenging. With more research and exploration, a safe and minimally invasive approach, along with selective and effective decompression, will enable successful surgery.

### Acknowledgement

This research was supported by a Korea University Medical Center Grant.

### Author contributions

N. Kim analyzed and interpreted the patient data. S.B. Jung performed the clinical examination and surgery, and was a major contributor in writing the manuscript. All authors read and approved the final manuscript.

## Supplementary Material


